# Full Characterization of Corpus Luteum Morphological Dynamics, Echotexture, and Blood Flow During Different Stages of the Follicular Wave in Spontaneously Non-Mated Female Camels (*Camelus dromedarius*)

**DOI:** 10.3390/vetsci12121212

**Published:** 2025-12-18

**Authors:** Abdulrhman K. Alhaider, Ibrahim A. Emam, Elshymaa A. Abdelnaby

**Affiliations:** Department of Clinical Sciences, College of Veterinary Medicine, King Faisal University, P.O. Box 400, Al-Ahsa 31982, Saudi Arabia

**Keywords:** luteal echotexture, Doppler, ovarian, ipsilateral, camel

## Abstract

This study was designed, for the first time, to fully characterize the corpus luteum’s (CL) dynamics, echotexture, and ovarian blood flow on the ipsilateral side of the CL during different stages of the follicular wave in spontaneously non-mated camels (*Camelus dromedarius*) and to correlate the CL’s size echotexture with Doppler parameters. Of 20 non-mated camels, only 7 exhibited spontaneous ovulation. B- and color-mode analyses of the CL were estimated; CL frozen image echotextures [CL echogenicity (CLE) and CL heterogeneity (CLH)] and ovarian artery (OV. A.) dynamics were recorded, and ultrasound scanning was performed. Blood sampling and progesterone (P4) levels were measured after ovulation. CL diameter and echotexture were elevated (*p* = 0.025 and *p* = 0.037) at the mid-maturation stage and compared to the early growth and late regression stages (1.03 ± 0.45/cm and 82.65 ± 2.87 for CLE and 33.65 ± 1.83 for CLH vs. 1.98 ± 0.88 cm; 66.52 ± 4.32 for CLE and 15.66 ± 0.25 for CLH vs. 1.02 ± 0.02 cm, 65.12 ± 2.66 for CLE, and 19.32 ± 1.33 for CLH), as those parameters are critical in the determination of CL activity. Ipsilateral OV. A. diameter increased (*p* = 0.021) in the mid-maturation and regression stages, with a significant elevation in Doppler velocities (*p* = 0.025) in the maturation stage, with a decline in Doppler indices (*p* = 0.013), while the contralateral side was not affected. Ipsilateral mean velocity (Vm; cm/s) and blood flow volume (BFV; mL/min) were increased in the mid-maturation stage (23.55 ± 0.66 cm/s and 25.62 ± 0.32 mL/min). CL diameter was positively correlated with the CL’s total colored area/pixels (r = 0.81; *p* = 0.001), total colored area % (r = 0.93; *p* = 0.001), and OV. A. velocities (r = 0.96; *p* = 0.001). In addition, there was a positive correlation between CLH and OV. A.BFV (r = 0.89; *p* = 0.001). After spontaneous ovulation, the CL increases in diameter and reaches its peak on day 12, with an elevation in the P4 level at day 10, and the total colored area of the CL continues to elevate until day 14. Ipsilateral OV. A. blood flow is elevated and linked to changes that occur in the CL’s total coloration %. Evaluating luteal function in camels presents several challenges due to the species’ unique reproductive physiology.

## Simple Summary

The corpus luteum’s dynamics, echotexture, and ipsilateral ovarian blood flow during different stages of the follicular wave in spontaneously non-mated camels were examined. Ultrasound scanning, blood sampling, and progesterone levels were measured after ovulation. CL diameter, echotexture, and ovarian arterial Doppler parameters were elevated in the mid-maturation stage. After spontaneous ovulation, the CL increases in diameter and reaches its peak at day 12, with an elevation of P4 level at day 10, and the total colored area of the CL continues to elevate until day 14. This study demonstrated that evaluating luteal function in camels presents several challenges due to the species’ unique reproductive physiology and management factors.

## 1. Introduction

Camels are considered seasonal breeder animals with increased sexual activity at the breeding time from December to May in the winter season, with an induced ovulation type [1,2,3]. Since female camels are considered induced ovulators, their follicles have a tendency to regress during the early stages of growth and maturity [1,4,5]. When the dominant follicle that is ready to ovulate reaches a diameter of 0.9–1.9 mm, the proportion of ovulatory follicles drastically decreases [5,6]. Spontaneous ovulation can occur in camels in a regular cycle without stimulus, while in induced ovulation, camels require the mating process to trigger the hormonal threshold for the ovulation process [4,5]. Spontaneous ovulation was previously determined in cats and camels [7,8], with an incidence of 5–22% without any direct coital contact [9]. Some studies have documented the occurrence of spontaneous ovulation in camels [7,10] and llamas [11].

Corpus luteum (CL) forms from the mature ruptured follicle and produces progesterone (P4) to support pregnancy with a limited lifespan of about 10–12 days in case of absence of conception, after that an atresia occurs, and corpus albicans is produced [4,11]. By evaluating luteal morphology by ultrasound and P4 production, a relationship has been found between the CL morphology and function in non-pregnant camelids [4]. Repeated ultrasonographic examinations or plasma progesterone (P4) have been used to evaluate CL development in non-pregnant dromedaries [12].

Doppler technology his now utilized to determine CL functionality and vascular status in bovine species [13,14]. By analyzing the vascularization of the CL and the preovulatory follicle, it forecasts a cow’s fertility [15]. Luteal vascularization is the subject of several studies that monitor early luteal blood flow, demonstrate luteal function throughout the bovine estrous cycle [16,17], and evaluate reactions of luteal blood flow and function following oxytocin administration [18]. Since the CL is the female dromedary camel’s principal source of P4, the P4 plasma level stays extremely low (<1 ng/mL) during the follicular wave when mating and ovulation are not occurring [19,20,21]. Not enough research has been performed on the camel species’ temporal correlations between systemic P4 concentrations and B-mode CL echotexture and color Doppler variations in CL tissue.

Assessment of CL image analysis has been measured by CL echogenicity (CLE) and CL heterogeneity (CLH). These parameters are important indicators for assessment of CL function, differentiating it from other masses as well as evaluating early pregnancy health via CL analysis [22]. The amount of echotexture reveals the pattern of echoes that are formed by the tissue and, thus, provides key information for organ status [23].

Despite being a highly vascularized structure during diestrus, the CL undergoes a considerable decrease in luteal tissue volume during blood perfusion [24]. Several studies have employed color Doppler to characterize changes in the CL’s morphology and function over this time [25,26,27,28].

Surprisingly, luteal blood perfusion decreases before structural regression [27], and during luteolysis, the correlation between circulating progesterone (P4) levels and CL blood perfusion is stronger than the correlation between P4 level and CL area [28]. For this reason, the practical use of Doppler ultrasound to assess the CL activity by its functionality and vascularity in camels has provided important insights to determine changes in CL function. Until now, no present studies have considered the ovarian artery (OV. A.) at ipsilateral and contralateral sides to ovulation in spontaneously ovulated camels. In addition, no correlations were detected between CL diameters and ovarian flow. In addition, many present studies fail to correlate a connection between CL and Doppler parameters in spontaneously ovulation due to its small number of cases. Therefore, this current study was designed to determine the full characterization of corpus luteum dynamics, echotexture, and ovarian blood flow at the ipsilateral side compared to the contralateral one during different stages of the follicular wave in spontaneously non-mated female camels (*Camelus dromedarius*) and correlate the CL dimension with Doppler parameters and CL echotexture.

## 2. Materials and Methods

### 2.1. Animals and Location

Our study was performed on twenty healthy adult cyclic non-lactating, non-pregnant female camels (*Camelus dromedarius*) at the camel research center and camel clinic at Teaching Veterinary Hospital at King Faisal University, Department of Clinical Sciences (25°23 N 49°36 E) ALHASA, Kingdom of Saudi Arabia, after receipt of acceptance and approval from the animal ethical committee at Deanship of ethical review at King Faisal University [KFU-2025-ETHICS3789]. This current study was conducted in the normal breeding season in camels from November 2024 to January 2025, with temperatures expected to be mild, with days around 25 °C in the morning, and about 11 h of daylight. The animals’ ages were about 7–10 years, and they weighed about 500–600 kg, with a body condition score of about 3.5. All females were free from any gynecological disorders or any cardiovascular diseases that affect the heart, according to their Doppler reading results. Animals were kept under optimum feeding and management conditions with a daily ration containing 7–10 kg dry matter and 20 kg fresh pasture and water ad libitum [29].

### 2.2. Experimental Design and Diagnosis of Spontaneous Ovulation

All camels were previously examined for two cycles, as each cycle takes about 3 weeks, with a normal ultrasonographic appearance of ovarian structures; therefore, the examination was about 6 weeks, and those that showed spontaneous ovulation were measured and examined in our study. Of the 20 female camels, only 7 exhibited spontaneous ovulation. All camels had experienced parturition within 1–2 years before this study, and all females had no contact with males (visual or olfactory). There was an old bull, about 10 years old, who was present at a distance of 5 m from the females, beyond solid walls between pens that blocked both sight and smell to ensure isolation between male and female. The male was not in a rutting period and showed no interest in females.

Ovulation was confirmed by the disappearance of the dominant follicle that emerged from the follicular wave with a diameter of more than 1.1 cm, which is called the major follicular wave. If a central cavity was present after this day (day of ovulation = day zero), it was now included in CL diameter, as previously determined [4].

CL activity and functionality were determined by progesterone (P4) level analysis and Doppler ultrasound of the OV. A. that supplies the luteal tissue. The luteal regression was performed after the maturation stage of CL. CL diameter was measured after ovulation via growth (1–5 D), maturation (6–10 D), and regression (11–16 D) stages after emergence of the follicular wave as determined in cows [30]. Diagnosis of spontaneous ovulation in camels is based on the following parameters: (1) disappearance of the dominant follicle of the new wave between examinations, (2) development of luteal structure after daily repeated ultrasonographic assessment, (3) elevation of P4 levels parallel with the presence of CL, and (4) no induced ovulation by any means.

### 2.3. B-Mode and Doppler Analysis of CL

**Figure 1:**
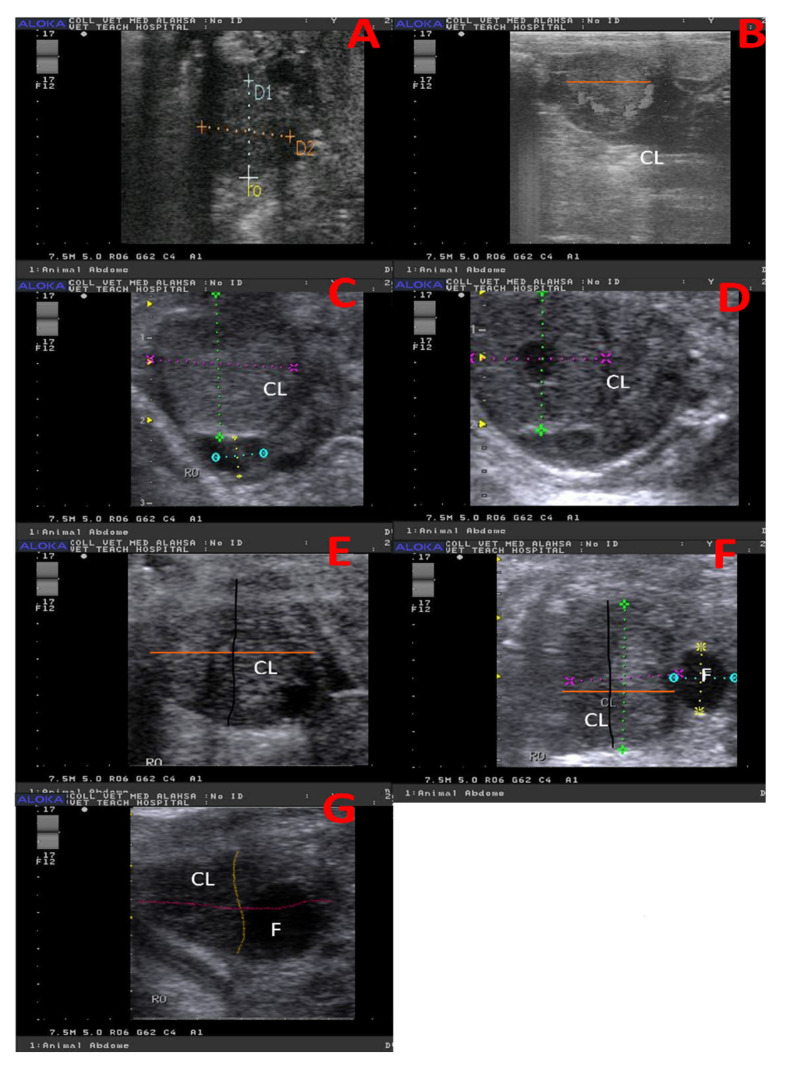
Ultrasound images (B-mode) revealed spontaneously formed corpus luteum diameter/cm at different developmental stages in a non-mated female camel (*Camelus dromedarius)* at day 2 (**A**), day 4 (**B**), day 6 (**C**), day 8 (**D**), day 10 (**E**), day 12 (**F**), and day 14 (**G**). F = follicle, CL = corpus luteum, and RO = right ovary.

**Figure 2:**
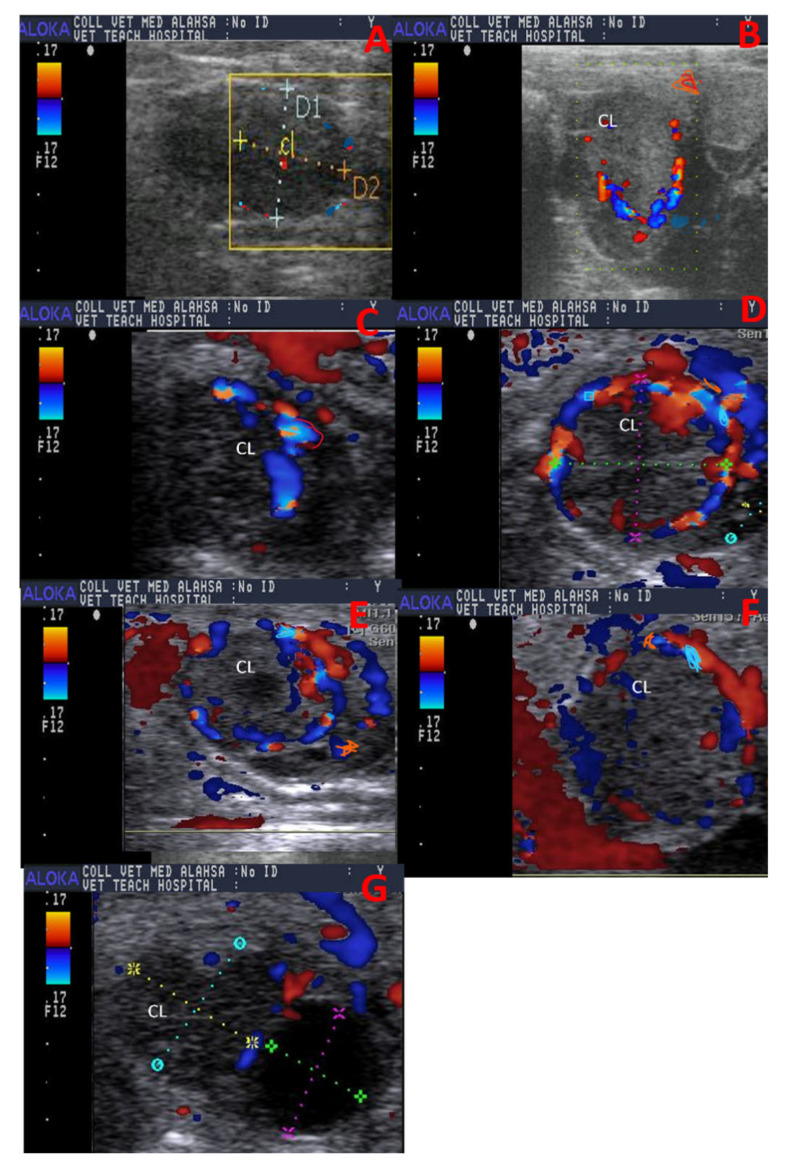
Ultrasound images (color mode; red and blue) revealed spontaneously formed corpus luteum colored area with a visualization of two colors [blue color which represent the direction of blood flow away probe toward CL and red color which represent the direction of blood flow toward probe away from CL] at different developmental stages in a non-mated female camel (*Camelus dromedarius)* at day 2 in the yellow box (**A**), day 4 (**B**), day 6 (**C**), day 8 (**D**), day 10 (**E**), day 12 (**F**), and day 14 (**G**) with measurement of new follicle appeared with a diameter in dotted lines of green and pink colors. F = follicle, CL = corpus luteum, and RO = right ovary.

CL dynamics and morphometry were measured using B-mode scanning using device ALOKA PRSOUND 3500SX (ALOKA, Tokyo, Japan) with 7.5 MHz rectal probe with gray, color, power, and spectral modes. After making fecal back racking, a linear probe was inserted in the rectum to determine ovulation at the ovary and growth of CL. Dimensions were measured in B-mode frozen image; the frozen image was utilized after that in the determination of CL echotexture. CL diameter was estimated in different developmental stages in camels as depicted in Figure 1. Color mode was activated after B-mode, and the same image in B-mode was presented in color mode to show the amount of coloration in two different colors (red and blue; [31,32]) at different developmental stages, as shown in Figure 2.

### 2.4. Imaging of CL Further Analysis

**Figure 3:**
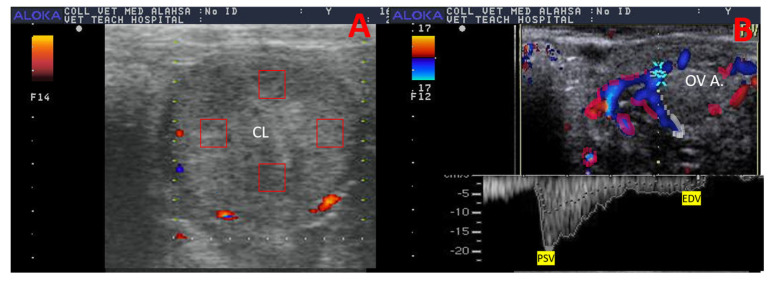
Ultrasound images (power mode frozen image) revealed 4 squares at (1 cm^2^) as red boxes in Adobe Photoshop program to determine the CL echotexture and measurement of echogenicity and heterogeneity (**A**). In addition, the ovarian artery appearance (**B**) by color Doppler mode was then pulsed wave mode was activated with an open gate window to determine the Doppler parameter. CL = corpus luteum, PSV = peak point of velocity (cm/s), EDV = end point of velocity (cm/s), and OV. A. = ovarian artery. Note this image was taken at day 9 after ovulation with the presence of tiny, smaller blood vessels that did not appear in color mode, as power mode was justified to show very small arterioles.

Firstly, CL echotexture was measured by CLE and CLH, as both parameters were measured by B-mode frozen CL image via Adobe Photoshop CC software (1990–2013, Adobe Systems) by drawing a 1 cm^2^ to determine the echotexture in the organ of interest (Figure 3A), as previously determined [33].

Secondly, the colored area in pixels and colored area % were also determined via Adobe Photoshop CC software (1990–2013, Adobe Systems) by using the color mode frozen CL image. Both blue, which represents blood flow direction away from the probe toward CL, and red, which represents blood flow direction away from CL toward the probe, represent two important colors that were analyzed and measured in the form of colored area/pixels and colored area % [34]. This color mapping showed blood flow direction in the area of interest, not oxygenation of the organ, with brighter colors representing a faster blood flow speed [35]. The measurement was performed by using the magnetic lasso tool to make the encounter of the colored area as determined in cows [16]. At the same time, the colored area % was determined by dividing the colored area by its total area [36,37].

### 2.5. Ovarian Artery (OV. A.) Morphometry and Hemodynamics

**Figure 4:**
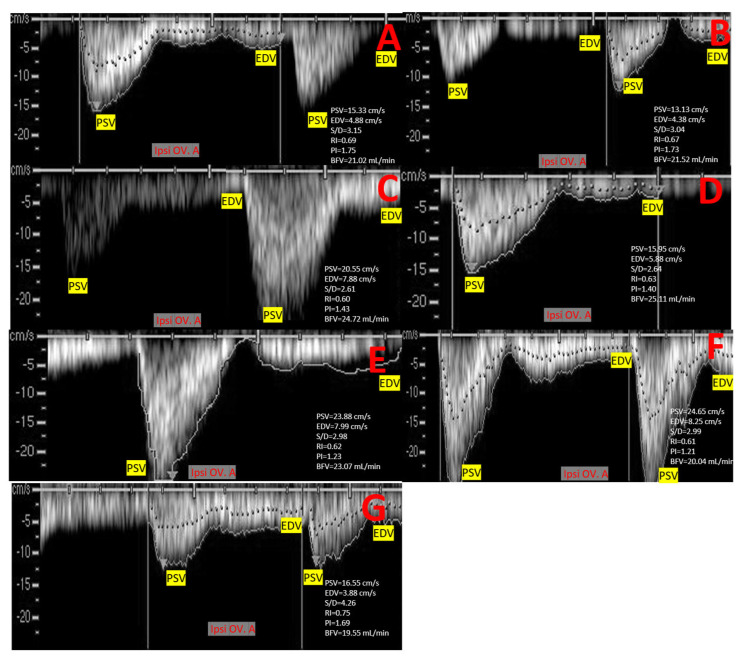
Ultrasound images (pulsed-wave mode; cardiac cycle graph) revealed ipsilateral ovarian artery (Ipsi. OV. A.) spectral graph to measure changes inside the artery by opened window gate to calculate Doppler parameters at different developmental stages in non-mated female camel (*Camelus dromedarius*) at day 2 (**A**), day 4 (**B**), day 6 (**C**), day 8 (**D**), day 10 (**E**), day 12 (**F**), and day 14 (**G**). PSV = peak point of velocity (cm/s), EDV = end point of velocity (cm/s), S/D = ratio between measured PSV/EDV. RI = resistance index (PSV-EDV/PSV), and PI = pulsatility index (PSV-EDV/TAMV). TAMV = time to achieve one complete cardiac cycle (cm/s). BFV = blood flow volume (mL/min).

After emerging from the abdominal aorta 3–7 cm before the external iliac artery origin, camel OV. A. is situated at the sixth lumbar vertebra [38,39]. Because of its tiny size, the OV. A. is difficult to identify by ultrasonography, yet detectable by color and spectral wave Doppler modes. OV. A. was estimated in different developmental stages by entering spectral mode and making an open window gate to measure Doppler parameters of interest in the ovarian artery ipsilateral to the CL formation (Figure 3B and Figure 4). The Doppler ultrasound settings were as follows: 40° angle of insonation, maximum velocity 40 cm/s, gain is 60 dB, brightness is 75%, gate size is 1 mm, region of interest (ROI) is standardized with only one operator, three-coloring map, and pulse repetition frequency was 4000 kHz [40]. The measured parameters were peak point of velocity (PSV; cm/s), end point of velocity (EDV; cm/s), ratio between measured PSV/EDV = S/D, resistance index (RI = PSV-EDV/PSV), pulsatility index (PI = PSV-EDV/TAMV), and time to achieve one complete cardiac cycle (TAMV; cm/s). Applying the identical blood vessel’s velocity and cross-sectional surface area, blood flow volume (BFV) was computed as follows [27]: blood flow volume (mL/min) = TAMV (cm/s) × π × (D in cm/2) where BFV is the blood flow volume (mL/min), and D is the diameter of the contralateral or ipsilateral ovarian or uterine arteries in cm [41].

### 2.6. Blood Sampling and Progesterone Analysis

CL diameter [42] and area [43] were related to progesterone (P4) levels. Blood samples were collected from animal’s jugular veins, and plasma was obtained after centrifugation at 1500× *g* for 10 min. Plasma samples were stored at −20 °C until hormonal analysis. P4 levels were measured in the ELSA kit (MyBioSource, UK, Competitive Type). Tests for inter- and intra-coefficient of variance were 10.7% and 12.4% with a number of technical replicates. Any value above 1 ng/mL is confirmatory for luteal appearance. The range of standards for hormones was 0.1–20.0 ng/mL.

### 2.7. Statistical Analysis

Our data were utilized as mean ± standard error of mean (SEM) using SPSS (Version 16.0, SPSS Inc., Chicago, IL, USA [44]). Data were first checked for normality using the Bonferroni test. A repeated measure ANOVA was used to show the day’s effect on CL diameter, colored area %, and P4 analysis [45]. In addition, ANOVA is also used to study the effect of stage (growth, maturation, and regression) on the following parameters (CL diameter, CLE, CLH, CL area/pixels, CL (upper, middle, and lower thirds) colored area/pixels, and total colored area/pixels with their colored area %). In addition, CL vascularization was determined by OV. A. (RI, PI, PSV, EDV, Vm, S/D, TAMV, and BFV) either on the ipsilateral or contralateral side to CL formation. The Pearson correlation coefficient was applied to show the connection between CL diameter and percentage of colored area with Doppler indices, as those parameters are positively or negatively correlated with CL morphology. Data are considered significant when *p* is less than or equal to 0.05.

## 3. Results

### 3.1. CL Diameter, Echotexture, and Colored Area at Different Developmental Stages of Follicular Wave

All the variants presented in Table 1 [CL diameter/cm, CLE (NPVs), CLH sdNPVs, CL area/pixels, CL UT colored area/pixels, CL UT colored area %, CL MT colored area/pixels, CL MT colored area %, CL LT colored area/pixels, CL LT colored area %, CL T colored area/pixels, and colored area %, during the mid maturation stage] had higher value than early growth; stage and late regression stage (*p* < 0.05). CL diameter/cm measured (1.03 ± 0.45 vs. 1.98 ± 0.88 vs. 1.02 ± 0.02 cm), respectively, in early vs. mid vs. late stages. CL echotexture was examined in form of echogenicity and heterogeneity, both CLE (NPVs) and CLH (sdNPVs) were increased (*p* = 0.037) in the mid maturation stage (82.65 ± 2.87 for CLE, and 33.65 ± 1.83 for CLH) when compared to early (66.52 ± 4.32 for CLE, 15.66 ± 0.25 for CLH), and late stages (65.12 ± 2.66 for CLE, and 19.32 ± 1.33 for CLH) as depicted in (Table 1). Area of CL was increased (*p* = 0.018) linearly starting from the early stage (3552 ± 10.65/pixels), mid (4685 ± 11.52/pixels), and then declined in the late regression stage (3395 ± 21.32/pixels) with the most elevation at the mid static maturation stage (Table 1).

The CL middle third colored area/pixels and area% were elevated compared to both upper and lower thirds in all three stages. Colored area/pixels were increased in the middle third (1832 ± 12.88/pixels) compared to other two thirds (1698 ± 15.32/pixels and 1702 ± 12.55/pixels) at the mid maturation stage (Table 1) and showed higher value (*p* = 0.038) compared to its value in other two stages (1832 ± 12.88/pixels in mid vs. 1566 ± 10.32/pixels in early vs. 1598 ± 11.33/pixels in late).

**Figure 5:**
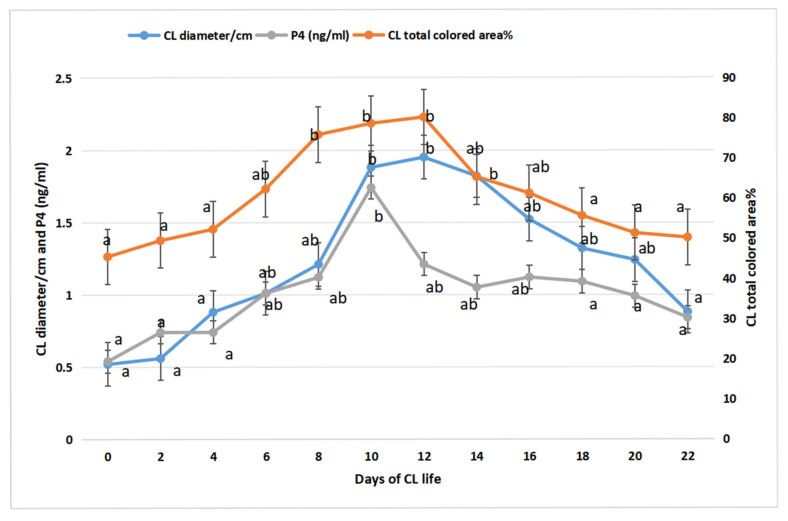
Alterations in CL diameter/cm and progesterone (P4; ng/mL) level analysis (primary axis), and CL total colored area % (secondary axis) at different developmental stages in non-mated female camel (*Camelus dromedarius*). Data are presented with error bars. Means with different superscripts showed a significant difference at *p* < 0.05.

Upper third colored area of CL and colored area% were increased (*p* = 0.039) in mid stage (1698 ± 15.32/pixels and 18.52 ± 1.55%) when compared to early (1352 ± 22.32/pixels and 13.21 ± 2.11), and late stages (1388 ± 10.55/pixels and 14.32 ± 1.32%) as shown in Table 1. Lower third showed the same pattern of elevation as upper third with more coloration area and more percentage as both colored area % and colored area/pixels were increased (*p* = 0.030) in maturation stage (20.12 ± 1.85% and 1702 ± 12.55/pixels) compared to other two stages (18.32 ± 0.47%, and 1366 ± 9.65/pixels in early; 17.32 ± 1.02% and1355 ± 14.32/pixels In late one). Finally, it is not surprising to obtain CL total colored area/pixels and their % with a marked elevation (*p* = 0.015) in the maturation stage (5032 ± 18.65/pixels and 73.32 ± 2.36%) compared to early (3512 ± 12.55/pixels and 56.32 ± 1.22%) and late stages (3992 ± 17.20/pixels and 55.92 ± 2.74%) as presented in Table 1. Total colored area % of CL was elevated (*p* < 0.05) gradually form day 0 after ovulation till reach a peak at day 12 the declined again till day 22 (Figure 5; secondary axis), while P4 (ng/mL) level was increased (*p* < 0.05) with CL diameter reach a highest level at day 12 after ovulation (Figure 5; primary axis).

### 3.2. Ovarian Artery Diameter, Velocities, Doppler Indices, and Flow Volume at Different Developmental Stages of Follicular Wave

Considering only the Ipsi variables (Table 2), the mid maturation stage was significantly higher than early growth and late regression stages (*p* < 0.05) for OV.A PSV (cm/s), OV.A BFV (mL/min), OV.A. RI, OV.A. PI, and OV. A Vm (cm/s). Valuating the ipsilateral and contralateral sides (Table 2), there was no significance in all the early growth stage variables (*p* > 0.05). There were significant differences (*p* < 0.05) in all mid maturation stages variables (*p* < 0.05), and in some late regression stages variables (*p* < 0.05) [OV.A diameter (mm), OV.A. PSV (cm/s), OV.A. EDV (cm/s), and OV.A. S/D].

Ipsilateral OVA. Diameter (mm) was increased (*p* = 0.021) in the mid maturation and regression stages (2.74 ± 0.01 mm and 2.71 ± 0.05 mm) compared to early stage (2.55 ± 0.02 mm), with a variation between ipsilateral side and contralateral one in the same stage. The contralateral OV. A. diameter was not changed (*p* = 0.314). Ipsi OV. A. PSV (cm/s) was linearly elevated along follicular wave with a marked significant increase (*p* = 0.025) at mid maturation stage (20.64 ± 0.32 cm/s) compared to early growth (15.36 ± 0.25 cm/s) and late regression ones (18.25 ± 0.65 cm/s), while the contralateral side of OVA. PSV was not affected (*p* = 0.554) as shown in Table 2.

In addition, OV.A. EDV was elevated (*p* = 0.034) in both maturation and regression stages (7.88 ± 0.52 cm/s and 8.02 ± 0.02 cm/s) compared to the early one (4.36 ± 0.04 cm/s) at the ipsilateral side, while no changes occurred in the contralateral one (*p* = 0.421). As a result of the elevation of PSV and EDV, this led to a decline in the S/D ratio, as this parameter was significantly (*p* = 0.028) declined in OV. A. at the ipsilateral side in maturation and regression stages (2.62 ± 0.05 and 2.24 ± 0.32).

Both Doppler indices expressed by RI and PI were significant declined because of the flow velocities at the ipsilateral side of OV.A. The most significant (*p* = 0.013) decline was observed in OV.A. RI at the maturation stage (0.61 ± 0.01) compared to the other. The same pattern of decline (*p* = 0.014) was observed in OV.A. PI at same stage of maturation (1.41 ± 0.01), while the contralateral side did not change in both parameters of Doppler indices. Both Vm (cm/s) and BFV (mL/min) at the contralateral side were not changed (*p* = 0.358 and 0.227), but ipsilateral side showed a marked elevation in maturation stage in both parameters compared to the other (23.55 ± 0.66 cm/s and 25.62 ± 0.32 mL/min), as shown in Table 2. CL diameter was positively correlated with CL total colored area/pixels (r = 0.81: *p* = 0.001), total colored area% (r = 0.93: *p* = 0.001), OV.A. PSV (r = 0.86: *p* = 0.001), OV.A. EDV (r = 0.96: *p* = 0.001), OV.A BFV (r = 0.85: *p* = 0.001) and was negatively correlated with OVA.RI (r = −0.91: *p* = 0.001), and OV.A. PI (r = −0.96: *p* = 0.001). CLE was correlated positively with OV.A.PSV (r = 0.77: *p* = 0.001), and OV.A.EDV (r = 0.88: *p* = 0.001). In addition, there was a strong positive correlation between CLH and OV.A.BFV (r = 0.89: *p* = 0.001).

## 4. Discussion

This is the first work to investigate CL dynamics and its blood flow with determination of vascular organ echotexture via image further analysis in different developmental stages of follicular wave in non-mated spontaneously ovulated she-camels. Similarly to our current work, the follicular wave in camels is classified as early growth, mid-maturation, and late regression stages throughout the animal breeding season, with a variation in the timing due to age and nutritional status [46,47,48]. As some studies reported that during the whole season, there was a follicular wave with a growth of follicles in both ovaries [5,49], CL size elevated in our study till it reached a peak at day 12 with a peak P4 level at day 10 in association with a peak of total CL colored area at day 14, then a slight regression occurred to all mentioned parameters.

Some previous studies have demonstrated the lowering of blood flow just after ovulation, and a marked elevation was noticed in order to share in the formation of new CL that started with blood clots and some granulosa cells, then mature CL [50,51] as observed in cows [52], mare [53], sheep [52,54], and goats [55]. Color signals are now presented that indicate the amount of blood flow entering the organ [56], which is considered a good indicator for organ functionality and activity, with confirmation of P4 secretion. In agreement with our results, Rawy et al. [6] showed the same elevation in the same species after using GnRH analogue. Other studies revealed that CL morphological alterations, such as size, are not related to physiological alterations such as P4 production and blood flow volume [57]. According to previous studies, CL functionality and activity are directly determined by its vascular tone more than its dimensions, as mentioned in llamas [58,59], bovines [27,32], and camels [16]. The linear temporary alterations occurring in the regression stage after maturation are a very important indicator for the organ luteolysis process [26], which is associated with vasoconstriction that led to a marked decline in colored area in three-thirds of CL and also a decline of total CL colored area/pixels with colored area%.

In our study, CL echotexture was elevated in the mid-maturation phase in the case of a mated spontaneously ovulated camel, when compared to early and late stages, as this parameter is expressed by two important measurements in the frozen image, making further analysis of CL of interest because CL is an organ with a mixed echotexture in ultrasound images, as there is a mixture between high- and low-reflection areas that represent the density (heterogeneity) and echogenicity of the examined organ [60]. CL heterogeneity is closely related to vascularization and fibrosis by dysregulation of the luteal microenvironment that led to changes in vascularization and the accumulation of fibrotic tissue [60].

This is mainly due to the presence of cellular (fibrin) and vascular (blood) components inside CL that reveal the organ functionality [61]. Therefore, mixed echogenicity that appears inside the CL with higher heterogeneity is very critical in organ functionality and in the production of P4 to calm the uterus [62]. This could also be reflected by an elevation of P4 on the same days with echotexture that confirmed our findings. This tool of examination could help in the determination of normal organ functionality and in the diagnosis of the hemorrhagic CL, which is accompanied by fluid or pain in camels [63]. In addition, it could help in the determination of CL wall thickness, which is directly related to the vascular nature of the functional CL.

To measure the changes that occur in CL, organs should be classified into thirds by Adobe programs for image analysis to make further colored area calculations and then measurements of total coloration with determination of coloration percentage [64]. The middle third of the CL was elevated in blood flow by counting the amount of colored area away from or toward the organ in our study; this could be due to the nature of the CL vascularization pattern [65,66]. The number of colored pixels in the middle third elevated in the newly CL, which met the same increase in P4 levels. This is not similar to other study showed a delay in the elevation of P4 about 4 days after the elevation of the colored area [6]. Similar studies in cows and heifers found an elevation of CL diameter at day 13, and the amount of colored area % was increased at day 12 [17,22,67]. Another study reported that the functionality of CL is more connected with its blood flow and Doppler velocities than with its size [18,68]. In agreement with other recent studies in veterinary practice, there was an inverse relation between both Doppler velocities and indices [69,70,71]. The luteal vascularization is elevated after spontaneous ovulation in the she-camel from day 1 after ovulation, and then declines at the luteolysis stage with a maximum elevation at day 14, which is in agreement with other studies also showing the peak at day 14 [17,27], day 11 [17,68,72], and day 12 [73]. Spontaneous ovulation in dromedary camels has not yet been conclusively shown for several reasons. Firstly, serum progesterone concentration is high for a brief period (4–5 days), and ultrasonographic morphology of the CL could be similar to that of large, luteinized follicles. For this reason, ultrasonographic examinations and blood collection for progesterone measurement are required daily, or two to three times a week, for a longer period to diagnose spontaneous ovulation and to exclude other options (luteinized follicles). Second, only non-lactating camels have been the subject of considerable research on follicular development [5,7]. Doppler ultrasonography could be a critical tool for differentiation between normal cyclic active functional CL and non-functional one [74]. Progesterone (P4) levels could help in differentiation between ovulatory and an ovulatory wave as previously determined [41]. In our study, P4 levels were elevated and reached their peak at day 10 after spontaneous ovulation, which is different from many other animals because camels are characterized by a short CL lifespan of about (8–10) days as presented by its levels in peripheral blood [75]; therefore, if the camel has not mated, P4 levels mostly remain lower [49].

Our current study reported the marked elevation in ipsilateral OV.A. PSV, EDV, BFV, and Vmean with a marked decline in both OV. A. RI and OV. A. PI with respect to S/D ratio. In addition, a negative correlation was observed between CL size and Doppler indices (RI and PI). This could be due to the main equation that led to measuring both RI and PI, as there was an inverse relation between both Doppler indices and blood flow parameters (PSV, EDV, and BFV), as previously estimated in many species [36,76]. Therefore, only ipsilateral side was markedly affected, and the contralateral one was not changed. This could be due to the vascular changes in the ipsilateral side, starting from newly vascular CL formation till its regression with vasoconstriction.

The linear decline in RI and PI of ipsilateral OV.A. indicated a higher vascularity to the organ with higher blood flow volume just after ovulation to meet the CL vascular demands in order to obtain bigger and perform accurate development [23]. The higher vascular perfusion to OV.A. could be reflected to the same side in the uterine artery [77], as both are also regulated by levels of hormones that led to elevation of vascular perfusion via vasodilatation, such as estradiol and nitric oxide [78]. The decrease in Doppler indices is associated with an increase in blood flow velocities that is associated with the elevation of arterial diameter, which may reflect an angiogenic factor that is responsible for CL formation in humans [79] and animals [80]. 

There are some limitations of this current study, such as animal movement, which can alter Doppler images due to artifacts. We get rid of this condition by waiting for animals to be calm to perform optimum measurement, as anesthesia could alter the blood flow reading and give a false result, in addition to a lack of inter-observer agreement metrics. Another limitation is a smaller sample size (n = 7); a small sample size could result in increased error with a lack of statistical power, as this could affect correlation data, but we could take the assessment with caution. Our future aspects were to show the whole cycle with the follicle at the follicular wave and show the amount of colored area in the best follicle with CL determination to give a full characterization of the camel cycle.

## 5. Conclusions

After spontaneous ovulation, the CL develops and reaches its peak in diameter at day 12 with an elevation of P4 at day 10, and the CL’s total colored area continues to elevate until day 14. Ipsilateral OV.A. blood flow is also elevated, linked with changes in CL total coloration %. Doppler ultrasound and organ echotexture could provide information regarding ovarian structure and could be used to predict any abnormal conditions.

## Figures and Tables

**Table 1:** Difference in CL diameter, echotexture, area, colored area, and coloration percentages in a non-mated female camel (*Camelus dromedarius*).

Variable	Follicular Wave			
	Early Growth Stage	Mid Maturation Stage	Late Regression Stage	*p*-Value
CL diameter/cm	1.03 ± 0.45 ^a^	1.98 ± 0.88 ^b^	1.02 ± 0.02 ^a^	0.025
CLE (NPVs)	66.52 ± 4.32 ^a^	82.65 ± 2.87 ^b^	65.12 ± 2.66 ^a^	0.037
CLH (sdNPVs)	15.66 ± 0.25 ^a^	33.65 ± 1.83 ^b^	19.32 ± 1.33 ^a^	0.013
CL area/pixels	3552 ± 10.65 ^a^	4685 ± 11.52 ^b^	3395 ± 21.32 ^a^	0.018
CL UT colored area/pixels	1352 ± 22.32 ^a^	1698 ± 15.32 ^b^	1388 ± 10.55 ^a^	0.039
CL UT colored area%	13.21 ± 2.11 ^a^	18.52 ± 1.55 ^b^	14.32 ± 1.32 ^a^	0.039
CL MT colored area/pixels	1566 ± 10.32 ^a^	1832 ± 12.88 ^b^	1598 ± 11.33 ^a^	0.038
CL MT colored area%	23.63 ± 1.66 ^a^	31.32 ± 0.84 ^b^	25.65 ± 2.51 ^a^	0.021
CL LT colored area/pixels	1366 ± 9.65 ^a^	1702 ± 12.55 ^b^	1355 ± 14.32 ^a^	0.030
CL LT colored area%	18.32 ± 0.47 ^a^	20.12 ± 1.85 ^b^	17.32 ± 1.02 ^a^	0.030
CL T colored area/pixels	3512 ± 12.55 ^a^	5032 ± 18.65 ^b^	3992 ± 17.20 ^a^	0.015
CL T colored area%	56.32 ± 1.22 ^a^	73.32 ± 2.36 ^b^	55.92 ± 2.74 ^a^	0.015

(^a^ and ^b^) Means with different superscripts showed a significant difference at probability (*p* < 0.05). CLE = corpus luteum echogenicity, CLH = corpus luteum heterogeneity, UT = upper third, MT = middle third, LT = lower third, T = total, NPVs = numerical pixel values, and sdNPVs =standard deviation of numerical pixel values. Data is presented as Mean ± SEM (standard error of the mean).

**Table 2:** Difference in ovarian artery diameter and Doppler parameters at ipsilateral and contralateral sides in a non-mated female camel (*Camelus dromedarius*).

Variable	Follicular Wave			
	Early Growth Stage	Mid Maturation Stage	Late Regression Stage	*p*-Value
Ipsi OV.A. diameter (mm)	2.55 ± 0.02 ^a^	2.74 ± 0.01 ^b^*	2.71 ± 0.05 ^b^*	0.021
Contra OV.A. diameter(mm)	2.51 ± 0.01	2.56 ± 0.01	2.54 ± 0.01	0.314
Ipsi OV.A. PSV (cm/s)	15.36 ± 0.25 ^a^	20.64 ± 0.32 ^b^*	18.25 ± 0.65 ^a^*	0.025
Contra OV.A. PSV (cm/s)	15.21 ± 0.01	15.65 ± 0.52	15.02 ± 0.63	0.554
Ipsi OV.A. EDV (cm/s)	4.36 ± 0.04 ^a^	7.88 ± 0.52 ^b^*	8.02 ± 0.02 ^b^*	0.034
Contra OV.A. EDV (cm/s)	4.25 ± 0.12	4.32 ± 0.02	4.39 ± 0.05	0.421
Ipsi OV.A. S/D	3.66 ± 0.01 ^a^	2.62 ± 0.05 ^b^*	2.24 ± 0.32 ^b^*	0.028
Contra OV.A. S/D	3.58 ± 0.02	3.61 ± 0.01	3.54 ± 0.02	0.357
Ipsi OV.A. BFV (mL/min)	21.69 ± 0.55 ^a^	25.62 ± 0.32 ^b^*	20.32 ± 0.54 ^a^	0.021
Contra OV.A. BFV (mL/min)	20.31 ± 0.55	20.17 ± 0.01	20.92 ± 0.21	0.227
Ipsi OV.A. RI	0.69 ± 0.01 ^a^	0.61 ± 0.01 ^b^*	0.71 ± 0.01 ^a^	0.013
Contra OV.A. RI	0.69 ± 0.02	0.69 ± 0.01	0.69 ± 0.01	0.121
Ipsi OV.A. PI	1.71 ± 0.01 ^a^	1.41 ± 0.01 ^b^*	1.68 ± 0.01 ^a^	0.014
Contra OV.A. PI	1.69 ± 0.01	1.66 ± 0.01	1.69 ± 0.01	0.091
Ipsi OV.A. Vm (cm/s)	15.55 ± 0.21 ^a^	23.55 ± 0.66 ^b^*	16.21 ± 0.85 ^a^	0.042
Contra OV.A. Vm (cm/s)	15.32 ± 0.88	15.24 ± 0.54	15.66 ± 0.32	0.358

(^a^ and ^b^) means with different superscripts showed a significant difference at probability (*p* < 0.05), while * means there is a significant difference between the ipsilateral and contralateral side. Ipsi = ipsilateral, OV.A. = ovarian artery, Contra = contralateral, PSV = peak systolic point of velocity, EDV = end diastolic point of velocity, S/D = ratio between measured PSV/EDV. RI = resistance index (PSV-EDV/PSV), and PI = pulsatility index (PSV-EDV/TAMV). TAMV = time to achieve one complete cardiac cycle (cm/s). BFV = blood flow volume (mL/min), and Vm = mean velocity. Data is presented as Mean ± SEM (standard error of the mean).

## Data Availability

The original contributions presented in this study are included in the article. Further inquiries can be directed to the corresponding author.
